# Supplementary material for: Select Porcine Elongation Factor 1α Sequences Mediate Stable High-Level and Upregulated Expression of Heterologous Genes in Porcine Cells in Response to Primate Serum
Source: Genes (Basel). 2021 Jul 7;12(7):1046. doi: 10.3390/genes12071046 (PMC8304002; doi:10.3390/genes12071046)
Supplement: Supplementary file 1 [file genes-12-01046-s001.zip › genes-1253412-supplementary.pdf]

Supplementary Materials

Table S1. Putative transcription-factor binding sites in the selected *pEF1α* promoters compared with those of the *hEF1α* promoter.

Promoter	DNA strand	AP1	Sp1	HSF	CEBP	GATA	NF-κB
hEF1α	+	3	38	-	7	-	3
	-	4	42	1	4	2	2
pEF1α	+	- (3)	49 (29)	- (1)	3 (4)	-	1 (4)
	-	- (1)	45 (26)	- (1)	3 (4)	1 (1)	1 (6)

AP1, activating protein 1; Sp1, specificity protein 1; NF-κB, nuclear factor kappa beta; HSF, heat shock factor; GATA, GATA transcription factor; C/EBP, CCAAT-enhancer-binding protein. The number of transcription-factor binding sites located in the extended region beyond the 5' end of the corresponding sequence of the *hEF1α* promoter are shown in parentheses.

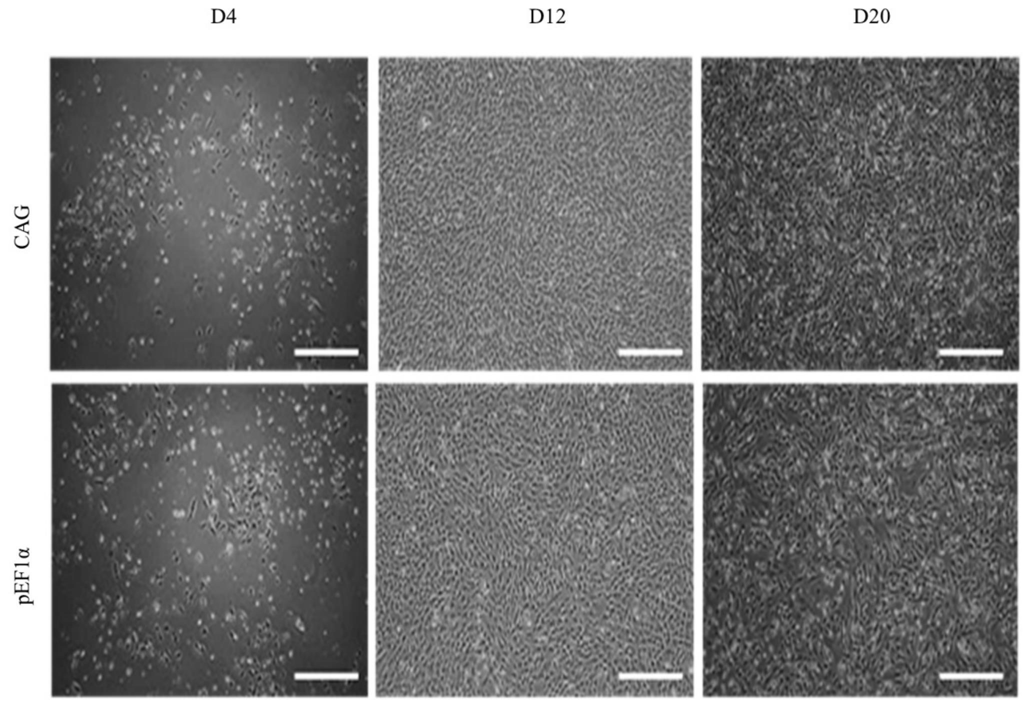

Figure S1. Morphological analysis of CAG- and the *pEF1α*-HLA-E expression cassettes transfected into PEFs and selected for on day 4 (D4), day 12 (D12), and day 20 (D20). The images revealed no changes in cell morphology. CAG, cytomegalovirus enhancer/chicken β-actin promoter; *pEF1α*, porcine elongation factor 1α. Scale bars indicate 200 μm.
